# One year of campaigns in Cameroon: effects on routine health services

**DOI:** 10.1093/heapol/czw054

**Published:** 2016-05-11

**Authors:** Sandra Mounier-Jack, Jean Marie Edengue, Mylene Lagarde, Simon Franky Baonga, Pierre Ongolo-Zogo

**Affiliations:** ^1^London School of Hygiene & Tropical Medicine, London, UK; ^2^Ministry of Health, Yaoundé, Cameroon; ^3^London School of Hygiene & Tropical Medicine, London, UK; ^4^Central Hospital, Yaoundé, Centre for Development of Best Practices in Health, Yaoundé, Cameroon

**Keywords:** Evaluation, health services, health systems, immunization

## Abstract

Background: Targeted campaigns have been reported to disrupt routine health services in low- and middle-income countries. The objective of this study was to evaluate the average effect of public health campaigns over 1 year on routine services such as antenatal care, routine vaccination and outpatient services.

Method: We collected daily activity data in 60 health facilities in two regions of Cameroon that traditionally undergo different intensities of campaign activity, the Centre region (low) and the Far North (high), to ascertain effects on routine services. For each outcome, we restricted our analysis to the public health centres for which good data were available and excluded private health facilities given their small number. We used segment-linear regression to account for the longitudinal nature of the data, and assessed whether the number of routine activities decreased in health facilities during periods when campaigns occurred. The analysis controlled for secular trends and serial correlation.

Results: We found evidence that vaccination campaigns had a negative impact on routine activities, decreasing outpatient visits when they occurred (Centre: −9.9%, *P* = 0.079; Far North: −11.6%, *P* = 0.025). The average negative effect on routine services [outpatient visits −18% (*P* = 0.02) and antenatal consultations −70% [*P* = 0.001]) was most pronounced in the Far North during ‘intensive’ campaigns that usually require high mobilization of staff.

Discussion: With an increasing number of interventions delivered by campaigns and in the context of elimination and eradication targets, these are important results for countries and agencies to consider. Achieving disease control targets hinges on ensuring high uptake of routine services. Therefore, we suggest that campaigns should systematically monitor ‘impact on routine services’, while also devising concrete strategies to mitigate potential adverse effects.

Key Messages

- The average effect of 1 year of campaigns on routine services was negative notably on outpatient services in the two regions of Cameroon surveyed and on antenatal care consultations (ANC) in the Far North region.
- Negative effect was more pronounced where frequency and intensity of campaigns was higher.
- With an increasing number of interventions delivered by campaigns and in the context of elimination/eradication targets, country and funders should systematically monitor ‘impact on routine services’, and devise concrete strategies to mitigate potential adverse effects of campaigns.

## Introduction

Mass campaigns have been used for many years in combination with routine health service delivery. They aim to support the control of diseases that affect a large part of the population, contribute to outbreak containment, respond to specific constraints such as remoteness, population attitudes and preferences, provide catch-up to supplement or enhance insufficient routine coverage rates and mitigate for inadequate existing infrastructure to deliver the routine intervention (Mills 2005; Dietz and Cutts 1997). They can be national or sub-nationally focused, deliver one or a combination of interventions and vary in their duration and the type of human resources involved. They have been used, in particular, in the context of diseases that are the focus of eradication and elimination programmes, and increasingly so for polio, measles and neglected tropical diseases. Over the years campaigns have sat at the heart of the debate between a vertical approach to health improvement that tends to provide the ‘solution of a given health problem by means of single-purpose machinery’ and a horizontal approach that invests in strengthening routine health services over the long term (Gonzalez 1965).

Several studies have highlighted the adverse effects that mass campaign can have on routine healthcare delivery (Cavalli *et al.* 2010; Coulibaly *et al.* 2008; Griffiths *et al.* 2011; Hanvoravongchai *et al.* 2011; Marchal *et al.* 2011; Mounier-Jack *et al.*, 2012; Verguet et al. 2012; 2015). All these studies were qualitative apart from one carried out in South Africa (Verguet et al. 2012; 2013). Few studies have sought to quantitatively assess the impact of mass campaigns on coverage rates. Most have focused on the effects of campaigns on routine vaccination. Analyses of polio national immunization days (NIDs) on routine coverage rates for instance were inconclusive (Aylward *et al.* 1997; Bonu et al. 2003; 2004). These studies were based on before and after household survey design and attribution for trends in routine coverage was often difficult to demonstrate. Two studies by Bonu *et al*, one from North India (2003) and one in sub‐Saharan Africa and South Asia (2004), concluded that there was no or only little evidence that NIDs led to a change in coverage of non‐polio vaccines. However, the latter study showed decreased vaccination coverage rates in four African countries during the NID period. More recently a quantitative analysis of vaccination coverage in South Africa indicated a significant decrease for eight health service indicators during a measles Supplementary Immunisation Activities implementation (Verguet *et al.* 2013).

All these studies have evaluated the effects of ***one***type of campaign [Polio or measles or Neglected Tropical Diseases (NTDs)] on health services although many report or comment that the average effects of the various types of campaigns are likely to significantly affect the routine of service delivery (Closser *et al.* 2014; Hanvoravongchai *et al.* 2011; Marchal *et al.* 2011).

In the current context in which African countries are conducting a high number of public health campaigns linked to eradication targets and availability of funding for these campaigns, there is a lack of understanding of the effects of such campaigns on routine healthcare services. There is currently no monitoring of routine health service activities during campaigns, and reporting traditionally focuses on daily completion of campaign specific targets. The recourse to campaigns will be compounded in the future by increasing investments by Gavi, the Vaccine Alliance, with the introduction of new vaccines requiring extensive catch-up campaigns for diseases such as Rubella, Japanese encephalitis and HPV (Gavi 2014), as well as recent calls for more mass drug administration to tackle NTDs (From Promises to Progress 2013).

The aim of this study was to fill this gap and assess the impact of public health and vaccination campaigns conducted over 1 year on the delivery of selected health services activities in Cameroon with a view to informing international public health community and country policy makers.

Cameroon was chosen for this study as it routinely conducts a variety of public health campaigns and uses a tailored sub-national approach depending on needs and epidemiological profile to design and implement campaigns. In 2012, its routine DTP3 coverage rate was 85% (WHO/UNICEF estimate). Its health service is predominantly public, in line with many countries in Sub-Saharan Africa. With a low density of healthcare professionals, in line with the vast majority of low- and middle-income African countries at <0.01 doctor and <0.5 nurses and midwives per 1000 population, we anticipate that such a country will be likely to be affected by campaigns (WHO^ 2014^). The number of campaigns in 2011 Cameroon was between 5 and 9 depending on regions (Table 1), which was not as high as other countries in the region such as Mali (14 campaigns in 2011) whose health system is reported to be weaker (Mounier-Jack *et al.* 2014).

**Table 1.** Public health campaigns in two regions Cameroon, 2011

Campaign	Geographic focus	Days	Intense	Dates
Polio NID, 1st round	Far North	3	Y	April 1–3
Polio NID, 2nd round	Far North	3	Y	September 30 to October 2
Child Health Week	Far North	4	Y	May 12–15
School de-worming	Far North	11	N	May 19–30
Neglected Tropical Diseases	Far North	60	N	June 25 to August 29
Child Health Week*	Far North	3	Y	November 18–20
Distribution of bed nets	Far North	6	Y	November 25–30
Meningococcal A vaccination (Menafrivac)	Far North	7	Y	December 6–12
Polio NID, 3rd round	Far North	3	Y	December 20–22
Child Health Week*	Centre	4	Y	May 12–15
School de-worming	Centre	8	N	May 19–27
Distribution of bed nets	Centre	5	Y	October 6 – 10
Neglected Tropical Diseases	Centre 14/30 DS	30	N	July 15 to August 15
Child Health Week*	Centre	5	Y	December 7–11

*Child Health Week involved distribution of vitamin A and Mebendazole for children between 6 and 59 months, and catch-up of routine vaccination for children <12 months of age and Tetanus Toxoid (TT) for pregnant women.

## Method

### Data

To evaluate the average impact of public health campaigns conducted over 1 year on the delivery of selected routine health services in Cameroon, we purposely selected two regions with different epidemiological and health systems profiles that translate into a different typology and number of campaigns over the period. Because of its proximity to Nigeria and Chad, the Far North region is a part of the Meningitis Belt and at additional risk of spread of Polio. This means that all campaigns in the Far North tend to be more focused on single diseases, whereas in the Centre, the focus is placed on a more integrated service delivery (e.g. Child Health Week). The Far North also traditionally receives more support from international agencies than the Centre. In addition, these two regions are the most populated ones in the country (Table 2).

**Table 2.** Characteristics of the selected study regions 2011 (Expanded Programme on Immunization 2011).

Region	Population	Number of health districts	Number of health facilities (public and private)	Number of public health facilities	Rate health facilities/ population	Coverage rate DTP3 (2011), %
Centre*	3,525,664	29	571	370	1/6174	82
Far North	3,480,414	28	308	260	1/11,300	84
Total Cameroon	19,406,110	2648	2648	1 888	1/7328	86

We categorized campaigns between ‘intensive’ and ‘non-intensive’ in order to reflect the demands of the campaigns on health service resources (e.g. staff). Intensive campaigns are concentrated over a limited duration (usually <7 days) and rely heavily on outreach strategies and draw on a high number and types of medical staff in health centres (Table 3).

**Table 3.** Organization of campaigns in Cameroon

Campaign	Target population	Campaign delivery strategy	Staff involved	Community health workers	Incentive paid to staff*
Intensive					
Meningitis A vaccination	9 months to 15 years old	Healthcare facilities, schools, outreach sessions	Nurse = supervision; medical doctor = supervision; midwives = vaccination; other staff = vaccinators or recorders	Yes	Yes
Bednets	0–5 years and pregnant women	Healthcare facilities, schools, outreach sessions	Nurse = supervision; medical doctor = supervision; other staff= census and distribution	Yes	Yes
NID Polio	0–5 years	Door to door, healthcare facilities, schools, outreach sessions	Nurse = supervision; medical doctor = supervision; midwives = vaccination; other staff= vaccinators or recorders	Yes	Yes
Child Health Week	0–5 years	Door to door, Healthcare facilities, schools, outreach sessions	Nurse = supervision; medical doctor = supervision; midwives = vaccination; other staff = vaccinators or recorders	Yes	Yes
Non-intensive					
Neglected Tropical Diseases	5 years and above	Door to door and outreach	Nurse = supervision; medical doctor = supervision; other staff= census and distribution	Yes (census, distribution & monitoring)	Yes
De-worming	5–15 years	School and outreach in community	Nurse = supervision; medical doctor = supervision; other staff= census and distribution	Yes, and teachers are also involved	Yes

*Note: Per diems amount to 2000 FCFA ($3.5) per day for NID and de-worming activities and 3000 FCFA ($5.2) per day for nursing staff for injectable vaccines (measles, MenA). Per diems for supervisors tend to vary between 5000 ($8.7) and 25,000 FCFA ($43) depending on the supervisor level.

### Sampling strategy

In each region, we randomly selected six health districts out of 29 in the Centre and 28 in the Far North. In each selected district, we randomly selected five health centres, bringing the total number of health centres surveyed to 30 in each of the regions (Table 2).

We collected daily routine activity data as specified in Table 4 in each health centre over a 1-year period from 1 January 2011 to 31 December 2011. The rationale for collecting daily data was to be able to detect the effects of campaigns—to do that we could not rely on monthly or yearly data that are indeed available, but had to obtain the adequate level of granularity in the data to be able to detect the effects of campaigns. Some campaigns only last for a few days (e.g. 5 or 7 days for the insecticide treated bednets distribution campaigns, even less for some others; Table 1). Other campaigns are held over two consecutive months (e.g. the NTD campaign), not to mention that campaigns occur almost every other month. Therefore, monthly data would have been useless in detecting the effect of campaigns because such data would have collapsed periods with and periods without campaigns, without any ability to distinguish them.

**Table 4.** Data collected

Service utilization outcome	Definition	Source
Vaccination	Total number of children vaccinated for DTP1, DTP2, DTP3+ all TT vaccinations provided to pregnant women	Immunisation register and tally sheets, monthly report children immunized
Antenatal	Total number of antenatal consultations ANC1+ANC2+ANC3	Antenatal register
Outpatient	All outpatient consultations	Outpatient register
Deliveries	All deliveries	Deliveries register

We used a pre-tested collection tool that manually retrieved data from health facilities’ activity specific registers. During the data collection, a supervisor in each region assured the quality of data retrieval.

### Data analysis

For the analysis, daily data of all health centres were aggregated for each region. When data from a given facility were missing for <5 consecutive days, we imputed the average utilization for the month. When data were missing for >5 consecutive days, the series for that particular outcome in that health facility was dropped from analysis (in general, data were missing either for entire months or just for random days).

As a result of this exclusion criteria, for the analysis of curative consultation, we excluded 4 health centres from the Centre region; 13 health centres were excluded for the analysis of antenatal care consultations (eight in the Centre and five in the Far North region) and 17 for the analysis of vaccinations (10 in the Centre and 7 in the Far North region).

To detect changes in utilization of services due to the implementation of the campaigns, we adopted the following segmented-linear regression approach:

$$Y_{t}=\;\alpha +\;\beta_{0\;}time+\;\beta_{1\;}campaign+\;\varepsilon$$

where $Y_{t}$ is the utilization outcome (Table 3) for day *t*, time is a continuous variable that captures the secular trend in utilization over the year, and campaign is an indicator variable that takes the value 1 when a public health or vaccination campaign happens on the day and 0 otherwise. A documentary review ensured that exact dates and duration of the various campaigns were established.

Therefore, the coefficient $\beta_{1\;}$captures the average change in daily utilization during the campaign. To give an indication of the size of this change, we calculated the effect size as a percentage change compared with the annual average utilization of the outcome of interest in the region. We report both the coefficient $\beta_{1\;}$and the percent change in the results.

The analysis was conducted using Stata 13.

## Results

Table 5 presents the number and characteristics of the health centres where routine data were collected. Overall the majority of centres surveyed were public and in rural areas, reflecting their share in the country outside the capital city Yaoundé.

**Table 5.** Characteristics of the original study sample

	Centre	Far North
Characteristics of health facilities sampled		
Urban	8	2
Rural	16	25
Total	24	27
Average daily visits per region		
Vaccinations	29.74 (*n* = 13)	50.81 (*n* = 21)
ANC	14.19 (*n* = 17)	28.35 (*n* = 22)
Outpatient	181.05(*n* = 21)	55.19(*n* = 27)

There was no evidence that campaigns had any negative impact on routine activities in private facilities, probably due to the limited power of the analysis considering the small number of private facilities. Results from our analysis include 51 health centres, all of which are public health facilities. According to the varying quality of data collected, we sometimes excluded some facilities from the analysis, doing this outcome by outcome (see criteria for exclusion in the methods section).

Tables 6 presents the impact of each individual campaign on outpatient consultations, antenatal consultations and the number of children vaccinated through the routine Expanded Programme for Immunisation (EPI).

**Table 6.** Impact (% decrease/increase) on the number of patients served

Type of routine service activity	Far North						Centre					
	Outpatient		ANC		Vaccinations		Outpatient		ANC		Vaccinations	
Type of campaign	%	*P* value	%	*P* value	%	*P* value	%	*P* value	%	*P* value	%	*P* value
Meningitis A vaccine	−24.7	0.095	−63.3	0.089	−71.2	0.064	Na		Na		Na	
Bednets	−29.5	0.06	−22.9	0.569	36	0.384	−3	0.92	27.8	0.53	17.7	0.738
De-worming	−13	0.067	2	0.906	9.8	0.589	−18.3	0.126	4.9	0.773	5.2	0.797
NID-Polio	−13.3	0.255	−67.7	0.034	−29.1	0.351	Na		Na		Na	
Child Health Week	−15.9	0.238	65.2	0.072	164.2	< 0.001	−28	0.303	−25.8	0.438	0	0.297

Na = the particular type of campaign was not conducted in the region.

Our analysis showed that negative effects of campaigns on utilization are primarily seen in the Far North region, where the range and intensity of campaigns is larger. The analysis reveals large drops in outpatient, antenatal consultations (ANC) consultations and routine activities during the resource-intensive Meningococcal A vaccination (MenA) campaign and a sharp decrease in both ANC visits and routine vaccination during the Polio campaign (Table 6). A significant negative effect of the bednets distribution campaign is seen on outpatient activities in the Far North but not on other types of activities (Table 6). Child Health Week results in a decrease of ANC consultations. The strong increase of routine vaccinations in the Far North reflects in fact that Child Health Week involved a planned catch-up of routine vaccinations as a part of the package of activities implemented (Table 7). More interesting is the fact that such an increase is not observed in the Central region where a similar catch-up was planned.

**Table 7.** Estimated average effect of public health campaigns on service utilization

	% reduction in outpatient visits	% reduction in ANC visits	% reduction in Vaccinations
Centre			
All campaigns	−9.9	0.2	−1.2
*P* value	0.079	0.910	0.815
Far North			
All campaigns	−11.6	−20.3	10.9
*P* value	0.025	123	0.427
Intensive campaigns (a)	−18.0	−70.4	22.2
* P* value	0.020	0.001	0.286
Intensive campaigns (except CHW)			−47.9
* P* value			0.051

Note: Intensive campaigns include MenA vaccination, Polio NID, Bednet distribution and Child Health Week (CHW).

Overall, the analysis shows that there is evidence that campaigns have a detrimental impact on the number of outpatient visits across the whole year (Centre: −9.9%, *P* = 0.079; Far North: −11.6%, *P* = 0.025), which means that more patients would likely have been seen in consultations if campaigns had not disturbed routine activities. If we consider intensive campaigns in the Far North that require a high concentration of activities and a strong involvement of staff over a few days and active outreach to the target population (Table 3), the overall effect of a 1-year campaign amounts to a significant drop of 18% of outpatient visits (*P* = 0.020), 70% of ANC visits (*P* = 0.001) and 47.9% of routine vaccinations (*P* = 0.05), once we have removed the effects of Child Health Week, which involved by design additional catch-up of unvaccinated children (Table 6).

Our analysis reported no effect on the level of deliveries, most likely due to the small number occurring in public health facilities. We found no significant differences between urban and rural facilities.

## Discussion

This is the first study to look at the average effects of public health campaigns on routine service utilization over 1 year. In one of the two regions with a higher intensity of campaigns, we reported a significant decrease of routine activities such as outpatient consultation, ANC and routine vaccinations. In the other region where few campaigns were implemented and some of these campaigns were delivered in an integrated manner, the average effect of campaigns could not be demonstrated, with the exception of a decrease in outpatient activities. We found that intensive campaigns that mobilize staff over a few days and involve outreach strategies such as MenA vaccination and polio campaigns or bednet distribution tend to result in a larger drop in activities, notably outpatient activities that decreased by 18% during campaign days in the Far North region. In the Far North, the negative impact of intensive campaigns is particularly striking for ANC visits (*P* < 0.001) with a reduction of 70.4% compared with average non-campaign days. We can hypothesize that this is because the nurse in charge of ANC is traditionally tasked with the role of supervisor during this type of campaigns. The positive impact of Child Health Week for catch-up vaccination drop-outs, and defaulters seem to have a larger positive effect in the Far North than in the Centre region although this activity was similarly included in the combination of services provided. A regional EPI post-campaign evaluation report indicates that targets for the vaccination of defaulters were not achieved in the Centre region, possibly indicating a lower quality of campaign and staff mobilization (Expanded Programme on Immunization 2011). Differences in terms of the impact of campaigns between the two regions could also be a reflection of the predominance of public health facilities in the North whereas in the Centre patients may be presented with wider opportunities to access services in neighbouring private health facilities. This difference could also the result of better organized and resourced campaigns in the North, which is traditionally prioritized by international organizations and NGOs. Finally, we cannot exclude that this could be a reflection of poorer quality of data for routine vaccinations in the Centre region (11 centres were excluded from the analysis in the Centre against 6 in the North).

Although campaigns decrease utilization of routine services, it can nevertheless be argued that they benefit the health system by providing effective interventions, in particular for hard-to-reach groups and remote areas and raise coverage thus decreasing disease prevalence and the need for possible outbreak responses. Catch-up of children for routine vaccines observed in the Far North may also contribute to increasing immunization coverage rates although the additional cost of the Child Health Week to the programme would need to be considered. Others also point to the fact that campaigns lead to the mobilization of additional resources that would not be available otherwise (Dietz and Cutts 1997).

Our study did not aim to dismiss campaign as a delivery strategy but investigate the possible average effects of campaigns, in a context in which each individual disease control programme tends to narrowly focus on its own campaign results, often dismissing a wider systems impact. Although the results we report are consistent with evidence from previous qualitative studies (Cavalli *et al.* 2010 ; Mounier-Jack *et al.* 2014; Closser *et al.* 2014), they highlight that the repeated occurrence of campaigns has an overall deleterious effect on basic service utilization. We suggest that as other have hypothesized (Hanvoravongchai *et al.* 2011) that lack of staff availability during campaign days reduces not only access during campaign days but also risks diminishing trust in services, reinforcing the poor image and low utilization of public health sector facilities (Gilson 2006). Researchers have also noted that the discrepancy between incentive systems of campaigns and routine activities distorts staff motivation and participation in the different types of activities (Fields *et al.* 2013). With some countries implementing more than one campaign every 3 weeks, the effects on routine services have become a major risk for the sustainability of routine health systems (Coulibaly *et al.* 2008 ; Heymann *et al.* 2010). Our findings also highlight a serious gap in policy: how to monitor and minimize these negative effects of campaigns.

Although this study was conducted solely in Cameroon, we argue that because of the great similarity between vaccinations programmes based on the expanded programme for immunization across African countries, as well as and other public health programmes, these are important and relevant findings for all countries that conduct a large number of campaigns. In fact at times many countries experience many more campaigns than Cameroon, and we can hypothesize that the effect that we found could be larger in many countries. This might be particularly relevant in the context of countries experiencing a high number polio immunization campaigns performed to achieve eradication (Closser *et al.* 2014).

Additional research is warranted to explore more specifically which healthcare workers are involved in campaigns with different delivery modalities, and how to re-balance financing and incentives of campaigns and routine activities; more importantly, more research is needed on designing and evaluating possible mitigating strategies such as a ‘minimum service’ during campaigns.

## Limitations

Only planned campaigns were included in this study. Localized campaigns conducted within outbreak responses were not included. Additional specific interventions are conducted in certain areas such as nutritional interventions like malnutrition screening, and these were not considered. This means that the effects detected on routine services are likely to be under-estimated.

Dates of campaigns used to conduct our analysis were restricted to the actual delivery of the campaign. In general, preparations for campaigns start several days (sometimes several weeks) before the actual start of service delivery activities, where healthcare staff undergo training and undertake a range of social mobilization activities. These preparatory activities are usually conducted by the head of the health centre, which means that we have probably underestimated additional disruptive effects to routine activities.

There are also limitations with data although efforts were made to triangulate data from different reports at health facility level (e.g. register, daily tally sheet and monthly reports). In the Centre region, where campaigns are less frequent, data recording was reported to be poorer and we had to exclude more facilities from our analysis as a result, notably of routine vaccination activities. This could mean that our findings might be weaker in the Centre region. Some data were missing and archiving practices varied, which led us to exclude some health facilities from our sample. It is unclear what the effect on our estimates is although it is likely that facilities with better and more organized record keeping are also more likely to be better staffed and organized to deliver services to the population. If this is true, it means that we will have under-estimated the effects of the campaigns.

## Conclusion

This study is the first that provide quantitative evidence of the average impact of repeated campaigns in one African country, Cameroon. We showed that critical services such as ANC and out-patients services were adversely affected by the nature and the number of public health campaigns. With many countries in Africa facing challenges to strengthen often weak health systems, we argue that it is time to re-balance investments in routine services away from one-off delivery campaign modes. We also suggest that because they are the main funders of campaigns, it is the responsibility of international agencies to ensure that their investments in campaigns are not at the detriment of routine activities. We argue that one way forward would be to systematically monitor routine activities during campaigns and introduce mitigating and rewarding strategies in order to maintain access to critical routine services.

## Ethical approval

Ethical approval to collect the data was obtained from the London School of Hygiene & Tropical Medicine. National ethical approval was not required for the study but administrative approval was secured from each the two regions’ health directorates.

## Funding

This study was funded by the Bill and Melinda Gates Foundation (Grant number OPP51822). 

*Conflict of interest statement*. None declared.
